# Lionel Shingleton Smith

**Published:** 1920-09

**Authors:** 


### LIONEL SHINGLETON SMITH.

The news of the death of Lionel Shingleton Smith, at the early age of 43, which occurred at Brecon on August 21st, came as a great shock to his many friends in Bristol. It was generally known that he had sustained a compound fracture of the leg as a result of a motor collision on July 16th, but such a tragic ending was unexpected. It was at first hoped that it would be possible to save the limb, but septic complications supervening, amputation was performed on August 9th. A temporary improvement only resulted, death finally resulting from pyæmia.

Lionel Shingleton Smith was the second son of Dr. R. Shingleton Smith, of Clifton, his younger brother, and fellow-student, Frank, having been killed on active service in Mesopotamia. Lionel was educated at Clifton College, St. John's College, Cambridge, and University College, Bristol, taking the degrees of B.A., M.B., B.C. (Cantab.), and the diplomas of M.R.C.S., L.R.C.P.

After qualification he held resident appointments at the Bristol Royal Infirmary, and was later House Surgeon to the South Devon and East Cornwall Hospital, Plymouth. At the termination of this appointment he set up in practice at Brecon, being appointed Honorary Physician to the Brecknock County and Borough Infirmary. Here he remained until death cut short his life's work.

A most lovable character, naturally of a cheerful and sociable disposition, Lionel Smith was a very popular student. That absolute unselfishness, which was the keynote of his character, endeared him to all with whom he came in contact. He always took the keenest interest and entered whole-heartedly into what was going on about him, whether work or play. Nothing was ever too much trouble for him, his patients from all classes finding him always ready and eager to do his utmost.

By his life of service and good-fellowship he has built for himself that most beautiful of all monuments, a place in the loving memory of his fellow-men.

Our deepest sympathy goes out to his widow and his four young children, to whom he was not only a father but a companion and playmate; and also to his father, Dr. R. Shingleton Smith, for many years the Editor of this *Journal*, in this his new sorrow.

A. J. W.

